# Self-Drying: A Gecko's Innate Ability to Remove Water from Wet Toe Pads

**DOI:** 10.1371/journal.pone.0101885

**Published:** 2014-07-23

**Authors:** Alyssa Y. Stark, Nicholas A. Wucinich, Eva L. Paoloni, Peter H. Niewiarowski, Ali Dhinojwala

**Affiliations:** 1 Integrated Bioscience Program, University of Akron, Akron, Ohio, United States of America; 2 Department of Polymer Science, University of Akron, Akron, Ohio, United States of America; Massey University, New Zealand

## Abstract

When the adhesive toe pads of geckos become wet, they become ineffective in enabling geckos to stick to substrates. This result is puzzling given that many species of gecko are endemic to tropical environments where water covered surfaces are ubiquitous. We hypothesized that geckos can recover adhesive capabilities following exposure of their toe pads to water by walking on a dry surface, similar to the active self-cleaning of dirt particles. We measured the time it took to recover maximum shear adhesion after toe pads had become wet in two groups, those that were allowed to actively walk and those that were not. Keeping in mind the importance of substrate wettability to adhesion on wet surfaces, we also tested geckos on hydrophilic glass and an intermediately wetting substrate (polymethylmethacrylate; PMMA). We found that time to maximum shear adhesion recovery did not differ in the walking groups based on substrate wettability (22.7±5.1 min on glass and 15.4±0.3 min on PMMA) but did have a significant effect in the non-walking groups (54.3±3.9 min on glass and 27.8±2.5 min on PMMA). Overall, we found that by actively walking, geckos were able to self-dry their wet toe pads and regain maximum shear adhesion significantly faster than those that did not walk. Our results highlight a unexpected property of the gecko adhesive system, the ability to actively self-dry and recover adhesive performance after being rendered dysfunctional by water.

## Introduction

The self-cleaning property of the adhesive toe pads of geckos has inspired and challenged material design of synthetics that are both adhesive and self-cleaning [1], [2], [3], [4]. The benefit to having a self-cleaning or an anti-fouling adhesive is clear. An adhesive that can clean itself, or avoid fouling all together, is likely one that can also be used multiple times and can be used on non-pristine surfaces such as those covered with dirt or dust. The self-cleaning behavior of the gecko's toes has two components. First, toes are cleaned by a passive self-cleaning method where dirt particles are more attracted to the surface a gecko walks on than the adhesive hairs, setae, which make up the small adhesive units of the toe pad [5], [6]. By lightly touching a dirty gecko toe to a clean surface dirt is removed and adhesion is recovered by 35.7% after eight simulated steps [6]. Recently however an active self-cleaning mechanism was also confirmed [7]. In active self-cleaning the peeling nature of the gecko toe via digital hyperextension helps expel dirt particles from the toe pads, significantly improving shear adhesion to nearly 80% of their original grip in only four steps [7]. The application of these findings are highly relevant to bio-inspired materials design, showing that after repeated use the fouled adhesive actually regains its adhesion rather than loses it. This recovery property is certainly not applicable for most pressure sensitive adhesives or commercially available adhesive tapes that can be easily contaminated [2], [4], [8].

Another innate and not entirely independent property of the gecko toe pad is its anti-wetting behavior. Similar to the self-cleaning property, gecko toes do not foul easily with water and although many synthetic adhesives either fail when used in water or after being exposed to moisture, the gecko toe pad is superhydrophobic and has a low contact angle hysteresis which causes water drops to bead up on a gecko toe and easily roll off without penetrating into the adhesive pad [9], [10]. In addition to cleaning dirt and water from the toe, we hypothesized that the anti-wetting toe pads should also allow the gecko to use its adhesive system in wet environments [11], [12]. As we found recently however, this is only partially true. In some instances a gecko toe can expel water trapped between the toe and a surface, but this is dependent on the thickness of the water layer [11], [13] and the wettability of the substrate the gecko clings to [12]. In fact, under certain conditions the toes can even lose their anti-wetting property [11], [12], [14]. For instance, we observed that geckos climbing surfaces wet with water droplets began to slip after running multiple times along that surface [11]. After inspecting their toes it was clear that the toe pads had become wet with water. We tested shear adhesion of wet toes to a dry glass substrate and found that even after taking four complete steps (involving digital hyperextension), shear adhesion was significantly lower (1.31±0.12 N) than geckos tested with dry toes (17.96±3.42 N) [11]. The results from this experiment show that even with the peeling action of four steps on a dry surface, similar to the active self-cleaning of dirt, geckos were only just able to support their body weight on a smooth glass substrate (∼1 N of force for a 100 g gecko), providing no safety factor for adhesion to the highly variable surfaces in their natural environment.

Previous studies highlight the gecko's retention of a high safety factor on smooth surfaces, approximately 20 times their body weight or more for a 100 g Tokay gecko (*Gekko gecko*) [11], [12], [15], [16], [17]. While there may be many reasons for the disparity between the necessary force to support body weight and the actual force available to geckos, wet toe pads may be one such factor. Many gecko species are native to tropical environments that experience high levels of atmospheric humidity and rainfall, likely wetting the surfaces a gecko moves across. While much effort has been focused on measuring the maximum adhesion geckos can obtain using dry toe pads on dry surfaces [15], [16], [18], [19], [20], an important question remains: how do non-functional toes that have become wet, perhaps after moving repeatedly across wet, rain soaked surfaces become dry and functional again? Is there some mechanism to enhance the removal of water and speed up the time it takes to regain adhesion? Contrary to the findings of self-cleaning dirt particles, our previous results do not show strong evidence for enhanced self-drying of toe pads that have become wet after four steps on hydrophilic glass (recovering only about 7% of their shear adhesion) [11]. Yet we expect geckos to encounter wet surfaces in many of their native habitats and to need to move successfully across them, which includes regaining adhesion after being fouled with water.

Although many studies focus on testing gecko adhesion on hydrophilic glass [11], [15], in their native environments geckos likely move across a diversity of surfaces, including those that are hydrophobic, like many plant leaves. The effect of wet toe pads on adhesion to a hydrophobic substrate has yet to be investigated but could help explain how geckos regain or even maintain functionality of their adhesive system in tropical environments where their toes can wet with water. Using the self-cleaning of dirt particles as an example, we hypothesize that active walking or stepping, using the gecko's unique stick-peel mechanism (digital hyperextension), will help to expel water from the toe pads and recover shear adhesion at a faster rate when compared to treatments when individuals are not allowed to step. Because the adhesive system is van der Waals-based [21], separation of the toe from the surface by a water layer can interrupt van der Waals forces and therefore we expect a hydrophilic glass substrate to be the least effective substrate for initial adhesion, as layers of water are more likely to remain trapped between the toe and the hydrophilic surface than mutually expelled by two hydrophobic surfaces (see[12]). As the gecko steps however, the hydrophilicity of the glass may help to pull water from the toe pads, recovering adhesion at a faster rate than a hydrophobic surface. To investigate both the effect of active self-drying (stepping) and substrate wettability on the recovery of shear adhesion after toe pads become wet, we tested geckos on a hydrophilic substrate (glass) and an intermediately wetting substrate (polymethylmethacrylate; PMMA) that we know geckos can adhere to underwater [12]. Geckos were either allowed to actively walk across the substrate prior to adhesion measurements or they were confined for a similar time period and not allowed to actively move across the testing substrate. Our results have significant implications for an improved understanding of gecko ecology, behavior and toe pad evolution, as well as for the novel design of a synthetic gecko-like adhesive that can recover functionality after becoming wet.

## Materials and Methods

**Figure 1:**
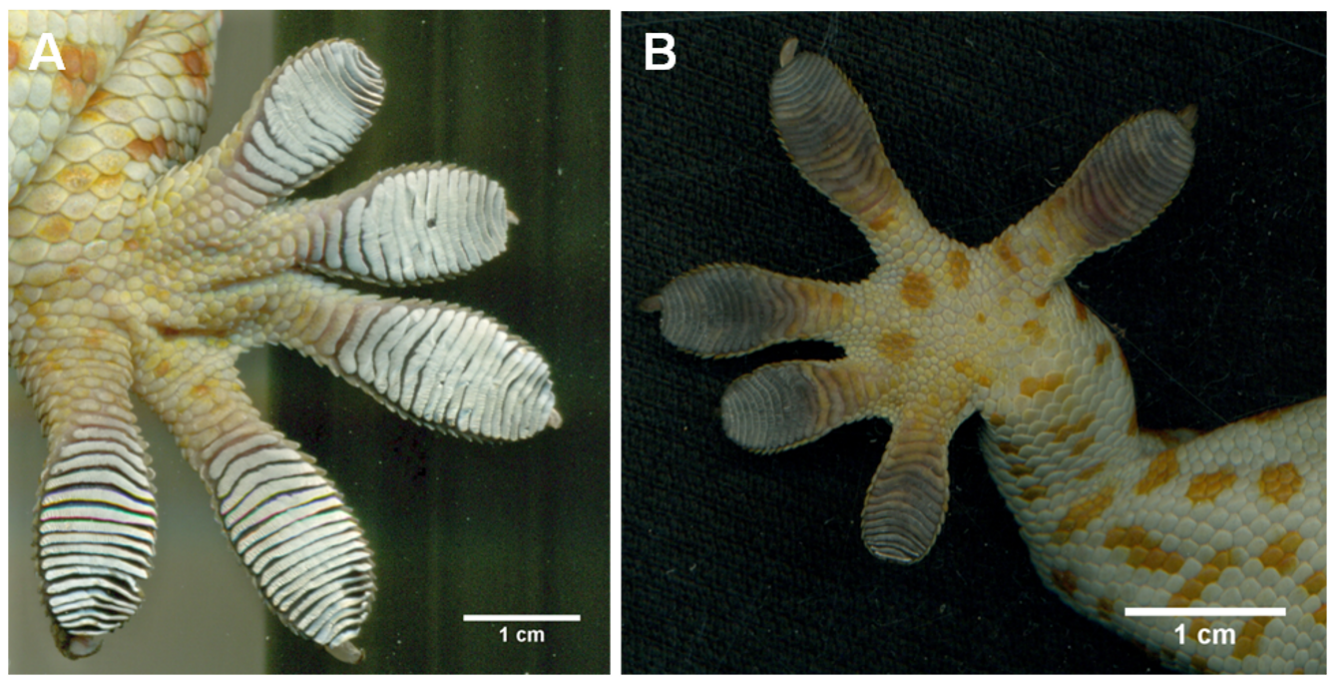
Wet and dry Tokay gecko (*Gekko gecko*) toe pads. (A) Dry foot in contact with a glass substrate where the setal mats appear white in color and (B) a wet foot in contact with a glass substrate where the setal mats appear grey in color. When wet the toe pads are no longer superhydrophobic and water droplets fall into the setal mat, completely wetting it.

Six adult Tokay geckos (*Gekko gecko*) were used for experimental trials. Geckos were individually housed as described in Niewiarowski et al. [15] and fed cockroaches three times a week and misted twice a day with water. Prior to experiments geckos were introduced to a walk-in environmentally controlled chamber that was kept at 24.2±0.1°C and 31.4±0.1% relative humidity for all experiments. After acclimating for at least 10 min, geckos were then acclimated to a foot soaking treatment. Foot soaking treatments were carried out similar to Stark et al. [11] where geckos were placed on a wet cloth and their toes were agitated to induce wetting of their toe pads for 11.0±0.3 min. Toe pads were visually inspected and confirmed to be completely wet (toe pads appear grey in color and are no longer superhydrophobic; see Figure 1A verses Figure 1B) prior to placing the gecko in standing water for at least 20 min. The 20 min time interval was chosen because a wetting transition in gecko setal mats appears to occur after 20 min of exposure to water [14]. After the agitation period, all geckos were placed in plastic tubs that had ∼0.5 cm deep water, enough to fully submerge their feet, and were soaked for 24.8±1.7 min. Water was kept at 21.9±0.1°C. After the 30 min total acclimation time (10 min pre-soak and 20 min soak with feet held underwater), geckos were then removed and their bodies were towel dried to remove excess water. Their toes were not touched but drip dried until water droplets stopped falling from their toe pads.

After soaking treatments geckos were either tested immediately or were induced to walk at least 10 steps with the front and at least 10 steps with the back feet on either an inclined glass or inclined polymethylmethacrylate (PMMA) substrate. Inclined substrates were used to induce digital hyperextension (toe peeling) [22]. Average number of steps for each treatment is reported below. When testing shear adhesion on a glass substrate, geckos walked along a hydrophilic glass substrate to self-dry. The same was true for the PMMA substrate. After testing shear adhesion the geckos were introduced to a small dry plastic box to prevent further stepping. Each gecko was tested at 0 min, 15 min, 30 min, 45 min and 60 min post-soak in succession, confined in the small box between timed adhesion tests. All six geckos were randomly tested in all experimental conditions which included adhesion tests on both glass (hydrophilic) and PMMA (intermediate wetting), with and without 20 steps prior to being tested at each time interval. Stepping substrates and adhesion testing substrates were cleaned first with ethyl alcohol and then water between each gecko. At the completion of each experimental trial geckos were weighed.

Shear adhesion was measured vertically using a custom rig as outlined by Niewiarowski et al. [15]. Geckos were outfitted with two pelvic harnesses and induced to take about four vertical positioning steps on the test substrate. Once all four feet had taken a step we moved a motorized force sensor at a controlled rate which pulled the gecko down the substrate via the harnesses that were attached to both the gecko and the motile force sensor. Maximum adhesion was measured as the point where all four feet begin to slide along the substrate. In some cases we found that damage occurred before all four feet began to slide, where strips of lamellae detached from the sliding toes. Because we were interested in the recovery time of shear adhesion, rather than maximum force, we outlined an experimental threshold for what we considered “time to maximum shear adhesion”. First, force values near or above 20 N of force were considered “maximum” based on our previous average forces on glass and PMMA [11], [12]. Once a force reading of near or above 20 N was recorded we stopped testing that individual and no further timed adhesion tests were completed, therefore in this scenario time to maximum shear adhesion was recorded for the trial where ∼20 N was reached. Second, we considered time to maximum shear adhesion to also be instances where damage to the toes occurred, even if this was below the 20 N threshold. When this occurred we also discontinued further timed testing and recorded the time where damage occurred as the maximum force the animal could sustain (to the point of damage). Finally, in some treatments, specifically the non-stepping glass treatment, neither “maximum” force (∼20 N) nor toe damage occurred after 60 min of testing, making this last time interval (60 min) our final cut-off for repeated timed testing. All procedures using live animals were approved by the University of Akron IACUC protocol 07-4G and are consistent with guidelines published by the Society for the Study of Amphibians and Reptiles (SSAR 2004).

We used a repeated measures MANOVA to test for an effect of substrate type (glass or PMMA) and self-drying treatment (stepping or no stepping) on time to maximum shear adhesion. Each gecko was tested under all combinations of treatment effects, removing the need to account for differences in toe pad area. To investigate time to maximum shear adhesion values of each treatment group we used a matched pairs analysis. Means are reported as mean ±1 s.e.m.

## Results

**Figure 2:**
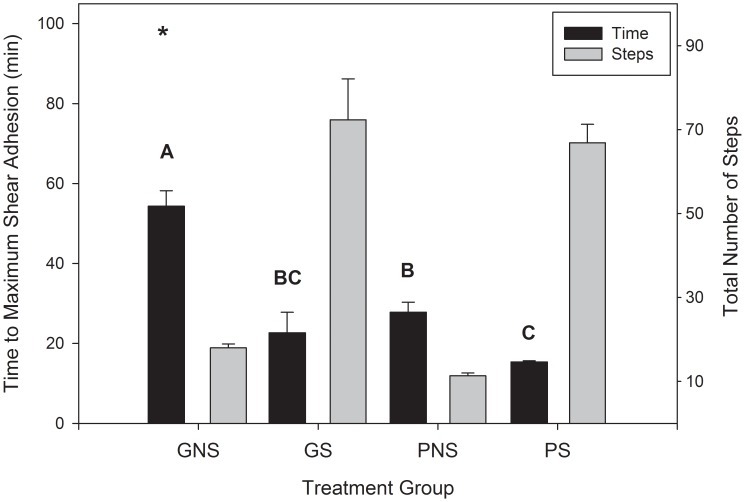
Time to maximum shear adhesion and total number of steps taken by Tokay geckos (*Gekko gecko*) with wet toe pads. Time to maximum shear adhesion (min) and total number of steps until maximum shear adhesion was reached for each treatment group (GNS  =  Glass non-stepping, GS  =  Glass stepping, PNS  =  PMMA non-stepping and PS  =  PMMA stepping). Bars with the same letter are statistically indistinguishable. Error is reported as mean ±1 s.e.m. The “*” represents the approximated time to maximum shear adhesion (min) in the GNS treatment group.

The average weight of the six Tokay geckos (*Gekko gecko*) during the experimental trial period was 102.87±3.30 g. In the self-drying stepping trials geckos stepped an average of 72±10 steps on the glass substrate and 67±4 steps on the PMMA substrate before the time to maximum shear adhesion threshold occurred, this includes approximately four steps to position themselves on the experimental apparatus. In the non-stepping group, where active self-drying was prevented, geckos were only allowed approximately four steps to position themselves on the substrate prior to adhesion testing. Geckos stepped an average of 18±1 positioning steps on the glass substrate and 11±1 positioning steps on the PMMA substrate in the non-stepping treatment groups before time to maximum shear adhesion was reached (Figure 2).

When testing for the effect of substrate (glass or PMMA) and self-drying treatment (stepping or non-stepping) on time to maximum shear adhesion we found a significant interaction between substrate and self-drying treatment (F_1,10_ = 9.54, p = 0.0115; Table 1). When geckos were not allowed to self-dry (non-stepping) and were tested on the glass substrate it took significantly longer to regain shear adhesion (54.3±3.9 min) when compared to when stepping on glass (22.7±5.1 min; t = −7.98, df = 5, p = 0.0005) and stepping on PMMA (15.4±0.3 min; t = −10.30, df = 5, p = 0.0001). The non-stepping glass treatment also took longer to achieve time to maximum shear adhesion than the PMMA non-stepping treatment (27.8±2.5 min; t = −7.28, df = 5, p = 0.0008). When we compared stepping on glass with the remaining groups we found that time to maximum shear adhesion did not differ from either the PMMA stepping treatment or the PMMA non-stepping treatment (t = −1.44, df = 5, p = 0.2094 and t = 1.03, df = 5, p = 0.3496, respectively). Finally, when tested on PMMA we found a significant difference in time to maximum shear adhesion between stepping and non-stepping treatments (t = −5.00, df = 5, p = 0.0041)(Figure 2).

**Table 1:** Repeated measures MANOVA shows a significant difference in time to regain maximum shear adhesion based on substrate (glass or PMMA), treatment (stepping or no stepping) and their interaction.

Effect	Wilks' lambda	Exact F	Numerator df	Denominator df	P value
Treatment	3.42	34.23	1	10	0.0002*
Substrate	2.94	29.43	1	10	0.0003*
Substrate X Treatment	0.95	9.54	1	10	0.0115*

## Discussion

Many previous studies have focused on the remarkable properties of the gecko adhesive system. It is self-cleaning, superhydrophobic, functional in water under specific circumstances, strong yet reversibly and directionally adhesive, reusable and virtually surface-insensitive [5], [6], [9], [12], [21], [23], [24], [25]. In this study we tested a new hypothesis: gecko toe pads are self-drying. It is counterintuitive that geckos from tropical environments routinely encounter wet surfaces which make their toes dysfunctional [11], yet have no way to regain their adhesion quickly. In response to this we found that active self-drying of toe pads occurs in Tokay geckos (*Gekko gecko*), and that substrate wettability does not have an effect on time to recovery.

**Figure 3:**
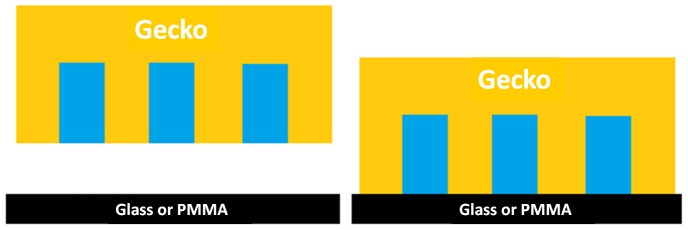
Schematic of the work of adhesion model geometry. Schematic depicts a patterned gecko surface (pattern of four setae represented as yellow pillars) filled with water (blue) both prior to and during contact with the substrate (either glass or PMMA) in air (white space).

When comparing stepping, or active self-drying, recovery times on either substrate (glass or PMMA) we found that stepping significantly quickened the time to regain maximum shear adhesion when compared to not stepping, allowing passive evaporation to dry the toe pads. This occurred on both substrates but the overall difference in time (non-stepping verses stepping) was larger when using glass as a substrate (difference of 31.7±4.0 min on glass and 12.4±2.5 min on PMMA). This supports our hypothesis that hydrophilic glass helps to wick away water more efficiently than a more intermediately wetting substrate like PMMA. Interestingly, while stepping on PMMA had the fastest recovery time (15.4±0.3 min), this did not significantly differ from stepping on glass (22.7±5.1 min). Therefore the time to maximum shear adhesion in active self-drying is not dependent on substrate wettability. When geckos were not allowed to step however, substrate had a significant impact on time to maximum shear adhesion, as geckos were able to regain adhesion through passive drying on PMMA faster than glass. This difference was large (a difference of 26.5±3.6 min) and it is important to note that in fact, half of the glass non-stepping group never reached the maximum force threshold (∼20 N or material failure) during our experimental trails. If we use the linear regression of force across time in the glass non-stepping group we can estimate that full adhesive recovery, assuming recovery rate is linear, will occur around 99 min, as shown as a “*” in Figure 2. This difference, of about 71 min, when compared to passive drying on PMMA is striking and shows that substrate wettability when passively drying (not stepping) has a strong effect on how quickly a gecko regains function of its adhesive system. It is not clear why active self-drying (stepping) is substrate insensitive and passive self-drying (non-stepping) is so clearly substrate sensitive, especially since we do not expect there to be a significant difference in surface roughness or amount of water initially held within the toe pad, which could contribute to differences in time to maximum shear adhesion. Using our previous work [12], we can explore this observation by considering the work of adhesion (W) to separate two surfaces (gecko setae and the substrate) in a direction normal to the surfaces when water is trapped between the setae prior to and during contact with either the glass or the PMMA substrates in air (Figure 3). We model the gecko setae as an oil-like surface (n-hexadecane) which is patterned in the shape of a tetrad (four setae) (see [12]). When this surface (h) makes contact with either substrate (s; glass or PMMA) water is held in the setal mat and does not interfere with the dry contact interface (an assumption of the model). Using Equation 1.1 we can predict adhesion between the two surfaces (h and s) where A_c_ is the total contact area (64 µm^2^) and A_2_ is the area of the substrate (121 µm^2^). Contact angles for the substrate (glass or PMMA) with n-hexadecane (θ_1_) and water (θ_2_) where measured elsewhere [12]. Finally, the surface energy of the gecko surface (h) in air (γ_h-air_ = 25 mJ/m^2^) and the surface tension of water (γ_air-water_ = 72 mJ/m^2^) are used. 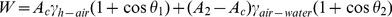
(1.1)


Using Equation 1.1 and reporting the work of adhesion as a ratio (W_PMMA_:W_glass_), we see that the work of adhesion ratio is 0.77, favoring adhesion on glass. Thus our thermodynamic model used here and previously [12], does not explain why geckos passively recover adhesion on dry PMMA faster than dry glass when water is trapped between the adhesive mats. This suggests that there are factors other than surface energies, such as water at the adhesive interface between the hairs and substrate, which causes passive adhesive recovery on glass to be much slower than PMMA.

**Figure 4:**
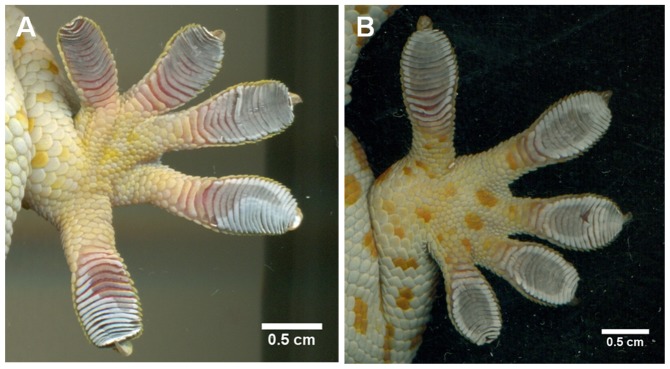
Tokay gecko (*Gekko gecko*) active and passive toe pad drying patterns. Appearance of toe pads at 15-soak in non-stepping (A) and stepping (B) groups. Areas that are grey in color are wet and areas that are white in color are dry. Without stepping toes heterogeneously dry, where some toes are wet and others show an irregular evaporation line (A). Conversely, when allowed to actively step toes dry in a more homogenous fashion, where the outside of the toe dries first, leaving a wet patch (grey in color) in the center of each of the toes (B).

Contrary to self-cleaning studies in beetles and passive self-cleaning in geckos [6], [26], our self-drying results show that over time and across steps adhesion is fully recovered. This is likely because water eventually evaporates in addition to being actively removed from the toes during active self-drying, whereas dirt particles can be trapped within the adhesive pads [6], [7], [26], [27]. Unlike self-cleaning of well defined dirt particles in laboratory studies, it is difficult to partition the contributions of evaporative drying and active removal of water via stepping. Clearly our results show active removal of water helps regain adhesion faster, but how? To observe differences in toe drying between the stepping (active drying) and non-stepping (passive evaporative drying) groups we imaged the toe pads at each of the early wetting intervals used for experiments. Initially toes in all groups were grey in color and clearly wet (Figure 1B) however after our treatment (stepping or non-stepping) we find distinguishable drying patterns at the next timed interval (15 min) (Figure 4). Here we see that geckos who were not allowed to take self-drying steps had variable drying patterns on their toes, where some toes remained wet and others became partially dry, shown by a patchy gray and white (wet and dry) appearance, often producing a clear evaporation line within a single toe (Figure 4A). Conversely in active self-drying by stepping we see well defined wet and dry regions of each toe where the perimeter of the toe dries first, leaving a wet patch in the center of all the toes on the foot (Figure 4B). This observation was made regularly in experimental trials. At the 30 min interval all toes appeared to be dry or nearly dry, where little grey color was observed and toes appeared qualitatively similar to a dry toe (Figure 1A). The difference in drying patterning is interesting because all groups were kept in small confinement boxes while waiting for the next timed testing interval, all being exposed to ambient evaporation, therefore the striking pattern difference in the self-drying group is clearly due to the active peeling mechanism of the toe.

In self-cleaning models we know that detachment of seta may help to actively and rapidly expel dirt particles from the toe [7], so perhaps a similar mechanism is occurring in self-drying. When dynamically self-cleaning dirt the toes are peeled distally, the setae separate in a fan-like manner and dirt particles jump off the setae [7]. If water droplets behaved similarly we would expect the toes to dry uniformly, however this is not the case. Likewise, if the fanning of the setae caused air to penetrate deeper into the setal mat for evaporation we would again expect no clear drying pattern. Instead we see an outer to inner medial pattern in the stepping groups. If we more broadly investigate the effect of morphological structuring on removal or transport of water however, there are several examples of structures in other organisms. Perhaps the more notable of these systems is the tree frog adhesive system. Tree frogs have a wet adhesive system where patterned microchannels hold fluid for use in capillary adhesion [28]. To retain the fluid, the channels use pressure differences to either move water out for adhesion or draw water back in for rapid removal of the toe and conservation of the adhesive liquid for the next step [28]. If we consider the wet gecko toe pad and the ordered array of setae, we can make the comparison to the tree frog toe pad where water is held between the setae of a wet toe and can be actively moved within the inner-setae and even inner-lamellar channels. Unlike the tree frog, we would expect movement of water out of the channels to be more highly emphasized than movement back into the gecko toe pad. This may be why frog micro- and macrochannels are hexagonally packed, which helps to move water in and out of the channels without removing it from the overall system [28], and why the gecko toe pad has channels that are linear, perhaps being used to direct water out of the toe permanently.

**Figure 5:**
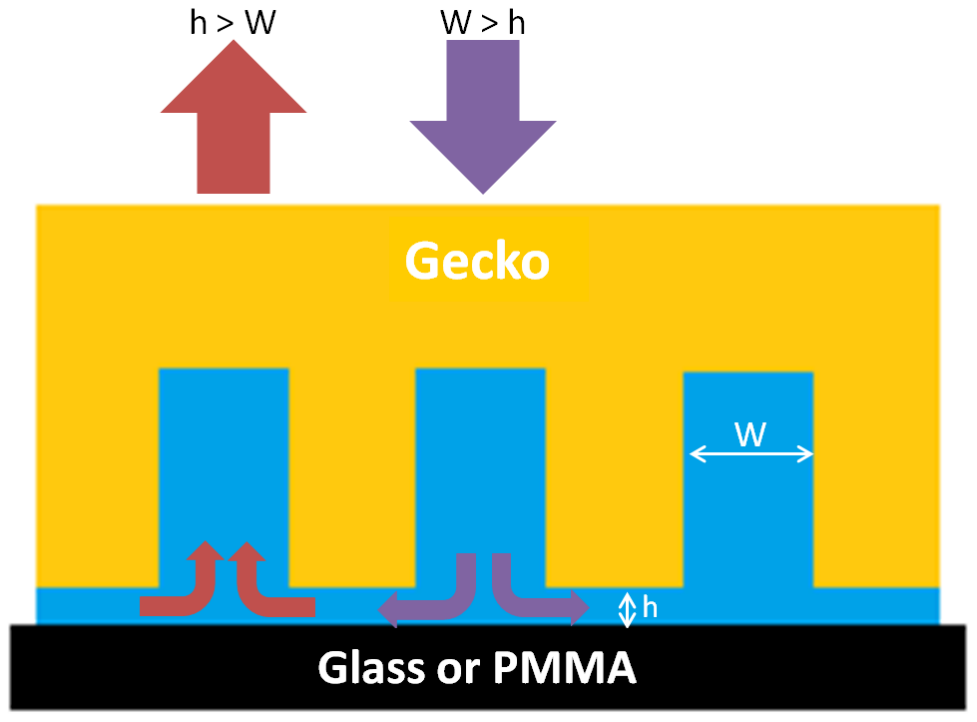
Schematic of the self-drying model. Schematic depicts a patterned gecko surface (pattern of four setae represented as yellow pillars) filled with water (blue) nearly contacting a substrate (glass or PMMA). Using the tree frog adhesive system as a model, we describe the inter-setal distance or microchannel width as W and the height of the intervening water layer as h. During a step, where the gecko setae approach the substrate, h→0 and W>h (purple arrow), causing water to move out of the microchannels (small purple arrows). When the gecko removes the foot, using digital hyperextension, h increases and at h>W (red arrow), remaining water is moved back into the microchannels (small red arrows). The movement of water in and out of the microchannels is due to the pressure difference in the microchannels and thin water film.

Using model predictions based on tree frog toe pads, we can roughly calculate the behavior of water in a wet gecko toe pad. Using the Tokay gecko (*Gekko gecko*) as our morphological model and only focusing on one level of hierarchy, the inner-setal regions, we estimate the spacing between the tetrad units to be about 2 µm [12]. If we consider this distance to represent the width of the microchannels (W) and h the height of the water layer between the toe and substrate, we find that W>h when the toe approaches close contact with the substrate (i.e. where h → 0). Estimation of the local pressure in both the fluid film and the inner-setal channels shows p_1_≈−γ/*r* (*r* = h/2) for the film and p_0_≈−γ/r* (r* = W/2) for the channel where the surface tension of water is γ≈0.07 N/m [28]. The difference in pressure of the channels and thin film is thus p_1_–p_0_<0 because W>h, therefore fluid flows from the channeled setal mats to the space between the toe and substrate. This pressure difference drives the movement of water out of the setal mats. To detach their adhesive toe pads both geckos and tree frogs use a peeling step which changes the pressure difference (p_1_–p_0_>0) at the point where h is greater than W. At this point (h>W) the difference in p_1_ and p_0_ causes water to be drawn back into the toe. A schematic (Figure 5) shows the microchannel width (W), height of the water layer (h) and both the application of the toe where W>h (in purple) and the retraction of the toe where h>W (in red), along with the direction of water flow for clarification. For tree frogs conservation of the water-lipid solution for the next adhesive step is advantageous, but for geckos it is clearly not and this could be why we see very defined patches of water in the toes pads after taking peeling steps, where water that was not completely removed from the toe-substrate interface is drawn back up into the setal channels at some critical peel angle where h>W. In groups that were not allowed to peel (step), drying patterns are more heterogeneous (Figure 4A) and thus are likely due to passive evaporation rather than active transport of water out of the toe pad by changes in pressure from the pressing and peeling of the toes in the stepping groups. Although it deserves further investigation, we hypothesize that when wet with water the inner-setal and even lamellar channels can act like the channeled treds on tires to help expel excess water from wet toe pads so that adhesion to the substrate can be regained more quickly than in a non-patterned surface.

Although the discovery of the self-drying mechanism and the observations we made have direct relevance for application to synthetics, the biological relevance of such a finding is also noteworthy. It is not entirely clear why adhesion is compromised when the toe pads of a gecko become wet but there are four possible explanations that may not be mutually exclusive. Generally, as expected, water layers between the setae and the substrate may cause the gecko to slip due to inadequate surface contact for van der Waals forces to occur. This occurs at the level of the toe with thick layers of water (∼0.5 cm) on glass [11], [12] and this may also occur between the setal tips, the spatula, and the substrate when the setal mats are permeated with water. We also know that at high levels of relative humidity (>80%) the setal modulus lowers [29], [30], which may impair the ability of the setae to orient and attach, especially when soaked in water for 30 min as was done here. Setae can also become self-matted when the modulus lowers or capillary forces draw them together, again limiting attachment and adhesion. Finally, two studies have reported that surface chemistry of the setal mat changes in some way that has yet to be fully understood when in the presence of water [13], [14]. Our results here suggest that self-matting can be reduced or even eliminated by standing on the toes, as there is no clear evidence of matting in the toe pads when in contact with a substrate (Figure 4) and that any changes in surface chemistry or modulus of the material is reversible over a relatively short timeframe (∼15 min for adhesive recovery). Therefore it is most likely that actual removal of water is most critical for regaining adhesion after toes have become wet.

To our knowledge there exists little evidence of self-drying in the natural world. Insects, including beetles, and tree frogs, use capillary adhesion and thus self-drying would be detrimental. For the dry adhesive system of the gecko however this appears to be imperative. But how can we relate these controlled laboratory tests to how geckos may utilize this unique property in their natural environments? First, we found that actively stepping significantly reduces the time it takes to regain maximum shear adhesion on either of the two substrates used. But how much distance is necessary to regain adhesion? If we estimate that Tokay geckos (*Gekko gecko*) can run 1 m in 3–4 strides, where one stride is two steps [31] the self-drying groups took 30–40 strides on average, a distance of about 10 m. Conversely the non-stepping groups took 5–10 strides, a distance of only 1–2 m. While it is difficult to predict what geckos do in their native environments, it seems unlikely that geckos would move 10 m at any one time. What is interesting here however is that we did not test geckos running, but rather taking controlled steps on an inclined surface. First, the distance covered by walking is likely much shorter than that by running, in fact we estimate that controlled stepping reduces the estimated running distance by at least half and second, the dynamic process of running may enhance self-drying and further lower the distance needed to regain maximum adhesion. In addition to the dynamics of running verses walking, our previous results where geckos took four steps on a dry horizontally mounted glass substrate with wet toes [11] are not comparable to those in this study where geckos took four steps on dry vertically mounted glass with wet toes, suggesting orientation may also have a significant effect on self-drying. The difference in initial cling forces on vertical and horizontally mounted glass (0 N and 1.31±0.12 N respectively) suggests a gecko can cling better when sitting horizontally, likely due to the pressure of their body weight and gravity helping to expel water, than those attempting to cling vertically. Although it is difficult to observe how geckos behave in their native environments, our laboratory-based studies suggest hypotheses of potential behaviors geckos may utilize when exploiting their natural habitat.

While it is interesting to consider a gecko making behavioral choices about where to walk or run, for how long and in what direction, it is important to be reminded of the complexity of the system as a whole. Specifically, the impressive safety factor that geckos utilize will likely allow for negligible effects on overall adhesion when only one or two toes are wet and all others are dry. While the total number of wet toes can certainly vary in their natural habitats and change based on how they utilize their adhesive system (walk verses run), the high safety factor geckos use may allow them to self-dry one or two wet toes while using the others to sufficiently maintain adhesion. Interestingly, we have observed that once wet, gecko toes are much more likely to become wet again over some time period. This was also suggested by Pesika et al. [14] when observing setal patches. So while it is unlikely all the toes of a gecko get wet all at the same time, repeated wetting may be a significant problem for geckos. Thus active self-drying may be important not just for drying a newly wetted toe, but also to help remove water from the toe pad after repeated exposure. It is also important to note that ambient temperature and humidity can also play significant role in self-drying and this may be highly relevant to geckos living in the tropics where temperature and humidity levels are high and wet surfaces are more prevalent. Further studies should investigate self-drying in different temperature and humidity regimes, pairing species-specific environmental values to rate of self-drying.

In this study we tested if an active self-drying mechanism, similar to the active self-cleaning mechanism, can help geckos recover the adhesive function of their toe pads. Our results reveal a surprising new property of the gecko adhesive system, the ability to self-dry and regain adhesion after being fouled by water. To our knowledge there are few, if any, instances were an adhesive, especially a non-permanent reusable adhesive, can regain adhesion after becoming wet. While this finding can be used to help improve synthetic adhesives, it is also relevant to gecko biology and helps to provide testable predictions about how geckos utilize their adhesive system in their natural environments. Clearly the natural habitat of geckos poses a variety of challenges, and as such we highlight here yet another new property of the gecko adhesive system, the ability to completely repair functionality after being rendered useless by water, the ability to actively self-dry.

## Supporting Information

Data S1 **Experimental data used for analysis.**
(XLSX)
